# A tunable ferroelectric based unreleased RF resonator

**DOI:** 10.1038/s41378-019-0110-1

**Published:** 2020-01-13

**Authors:** Yanbo He, Bichoy Bahr, Mengwei Si, Peide Ye, Dana Weinstein

**Affiliations:** 1 Purdue University, West Lafayette, IN USA; 2 Kilby Labs, Texas Instruments, Dallas, TX USA

**Keywords:** Electrical and electronic engineering, Electronic devices

## Abstract

This paper introduces the first tunable ferroelectric capacitor (FeCAP)-based unreleased RF MEMS resonator, integrated seamlessly in Texas Instruments’ 130 nm Ferroelectric RAM (FeRAM) technology. The designs presented here are monolithically integrated in solid-state CMOS technology, with no post-processing or release step typical of other MEMS devices. An array of FeCAPs in this complementary metal-oxide-semiconductor (CMOS) technology’s back-end-of-line (BEOL) process were used to define the acoustic resonance cavity as well as the electromechanical transducers. To achieve high quality factor (*Q*) of the resonator, acoustic waveguiding for vertical confinement within the CMOS stack is studied and optimized. Additional design considerations are discussed to obtain lateral confinement and suppression of spurious modes. An FeCAP resonator is demonstrated with fundamental resonance at 703 MHz and *Q* of 1012. This gives a frequency-quality factor product $f \cdot Q = 7.11 \times 10^{11}$ which is 1.6× higher than the most state-of-the-art Pb(Zr,Ti)O_3_ (PZT) resonators. Due to the ferroelectric characteristics of the FeCAPs, transduction of the resonator can be switched on and off by adjusting the electric polarization. In this case, the resonance can be turned off completely at ±0.3 V corresponding to the coercive voltage of the constituent FeCAP transducers. These novel switchable resonators may have promising applications in on-chip timing, ad-hoc radio front ends, and chip-scale sensors.

## Radio Frequency Devices: Unconventional Resonators in Conventional Microchip Technology

A reinvention of memory technology from Texas Instruments has enabled the creation of high-efficiency, high-quality resonators. Microscopic radio frequency (RF) resonators vibrate at stable frequencies, a property that can be used in tracking time, communicating, sensing, and imaging. Technological advances are demanding smaller, more power-efficient and cost-effective components, but traditional resonators are reaching their theoretical limits. Dana Weinstein and her team from the United States’ Purdue University and Texas Instruments have developed a new resonator integrated into existing chip fabrication with no post-processing, and offers much higher quality factor than alternate devices made side by side with electronic circuits. In this new paper, the team discusses the optimization that produced these low loss vibrations, and how the resonance can be manipulated by adjusting the electric polarization of the device.

## Introduction

Tunable high-Q, small footprint resonators have great potential in both mature and emergent fields such as radio frequency (RF) components^1,2^ and communication^3^, timing^4,5^, sensing^6–8^, and imaging^9^. The demanding performance of these devices and systems, alongside requirements for miniaturization, lower power consumption, and lower cost, has pushed the limits on what conventional technology can achieve^10^.

The authors have previously demonstrated high-Q, unreleased resonators with field effect transistor (FET) electromechanical sensing, referred to as Resonant Body Transistors (RBTs)^11^ in standard CMOS technology at frequencies ranging from 3 GHz^12^ to 32 GHz^13^. While the *f*·Q products of these devices are record breaking, their return loss and bandwidth are restricted by the fundamental limits of electrostatic transduction, which provides modest driving force density. This is particularly evident in the case of planar CMOS technology (e.g. 32 nm SOI), where the electromechanical transconductance of a 3 GHz resonator is on the order of 100 nS^14^. The correspondingly high insertion loss (IL) of such a device makes oscillator and filter design challenging. It is therefore necessary to explore alternative IC integrated materials.

Ferroelectric materials have spontaneous electrical polarization which can be reoriented under an external electric field^15,16^. The hysteretic behavior of ferroelectrics has driven fundamental investigation^17,18^ and commercial development for integrated circuit (IC) memory and has sparked a class of devices termed ferroelectric RAM (FeRAM or FRAM)^19,20^. Ferroelectrics have an additional trait of piezoelectric response; the changing polarization within the ferroelectric material induces dielectric dipole moment and changes the lattice constant, generating internal stress in the material^21^. Commonly used ferroelectric materials include barium titanate (BaTiO_3_)^22^, barium strontium titanate (BST)^23,24^, lead zirconium titanate (PZT)^17,25^, lead titanate (PbTiO_3_)^26^, and hafnium dioxide (HfO_2_)^27,28^.

Texas Instruments (TI) E035 FeRAM technology has integrated ferroelectric PZT in the back-end-of-line (BEOL) process of their 130 nm CMOS technology^29^. Leveraging this IC platform, we can realize ferroelectric based CMOS-MEMS resonators with higher electromechanical coupling coefficient (k^2^) than their electrostatic counterparts. The boost in performance facilitates larger bandwidth filters, lower power oscillators, and higher frequency tolerance to fabrication variations. This paper reports on the first piezoelectric resonators designed in TI’s FeRAM process. The designs presented in this work offer a unique approach to seamlessly integrated MEMS in solid-state CMOS technology, with no modification to the standard IC process. These devices also mark the first implementation of unreleased resonators based on ferroelectric capacitors.

## Device design and fabrication

In order to define the resonance mode with highest *Q* and transduction efficiency, acoustic waves must be well confined vertically in the CMOS stack with stress maximized in the PZT layer within the ferroelectric capacitors. Analogous to optical waveguide design, this is achieved using acoustic waveguiding within the CMOS stack. Assuming an infinitely long translationally invariant structure, we first determine the dispersion relations of the CMOS stack to define modes restricted to propagation in the plane of the wafer.

The device is first divided into multiple periodic unit cells with lattice constant *a*. To impose lateral (*x*-direction) translational symmetry, the left and right boundaries are defined as Floquet periodic boundary conditions

1 $$\vec u_R\left( {\vec r} \right) = \vec u_L(\vec r) \cdot e^{ - i\vec k \cdot \vec r}$$

where $\vec u_L$ and $\vec u_R$ are the displacement field at the left and the right boundaries of the single unit cell. $\vec k$ is the wave-vector which, due to the periodicity of the lattice, can be determined from the reciprocal lattice with a periodicity of $\pi /a$. This corresponds to the lateral width of the first irreducible Brillouin zone in the reciprocal lattice.

Two-dimensional finite element analysis is then performed in COMSOL Multiphysics® to map these waveguided modes. By applying Perfectly Matched Layers (PML) at the top and bottom of the device, no reflections from these boundaries are considered, approximating an infinitely thick Si substrate and thick, acoustically lossy dielectric layer in the BEOL.

**Fig. 1:**
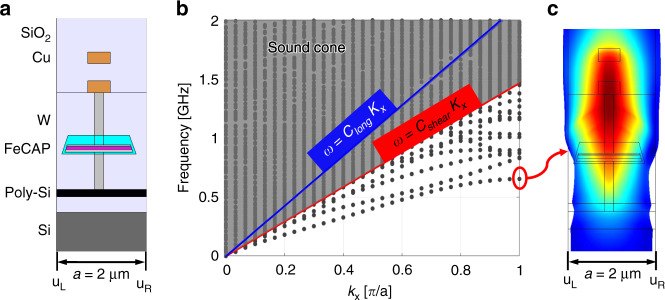
Dispersion relation analysis. **a** Schematic of a single unit cell. **b** Dispersion relation showing the vertically confined mode. Modes in the sound cone are free to propagate into the bulk Si substrate and cannot be well confined. **c** Corresponding displacement field of the localized mode shape of the unit cell FeCAP

Several designs are put forward and optimized. The details of the optimization process are summarized in the Supplementary Information. It is found that a device with trapezoidal FeCAP spanning 1.4 μm and two 0.6 μm wide metal layers above it exhibits the best vertical acoustic confinement. The corresponding schematics of a single unit cell is shown in Fig. 1a. The resulting dispersion relation for the acoustic modes in the CMOS stack is shown in Fig. 1b. Each point in the dispersion relation corresponds to an eigenmode of the periodic FeCAP structure. This dispersion relation can be divided into three regions separated by sound-lines (red and blue) represented by $\omega = c \cdot k$ where *c* is the corresponding longitudinal (blue) or shear (red) acoustic velocity in the Si substrate. Above the longitudinal sound-line is a region where all modes are free to propagate, referred to as the “sound cone”. Additionally, below the shear sound line exist several discrete modes with sufficiently low acoustic velocity, indicating that the elastic waves are prohibited from propagating in the bulk and the elastic energy is therefore confined in the BEOL region of the CMOS chip. The resulting solid-state acoustic waveguide assumes translational symmetry with a period of *a*. Any perturbation along the wave propagation direction will generate scattering, which will then result in the coupling of different waveguided modes and decrease the Q^12^. It was previously shown that driving along *k*_*x*_ *=* *π/a* is beneficial for reducing scattering to the sound cone, enabling higher *Q*^12^. In the meantime, the farther away the activated mode is from the sound line, the better confinement the resonator will achieve.

Fig. 1c shows a vertically confined mode at 700 MHz where the strain is well contained in the FeCAP, W via and Cu metal.

**Fig. 2:**
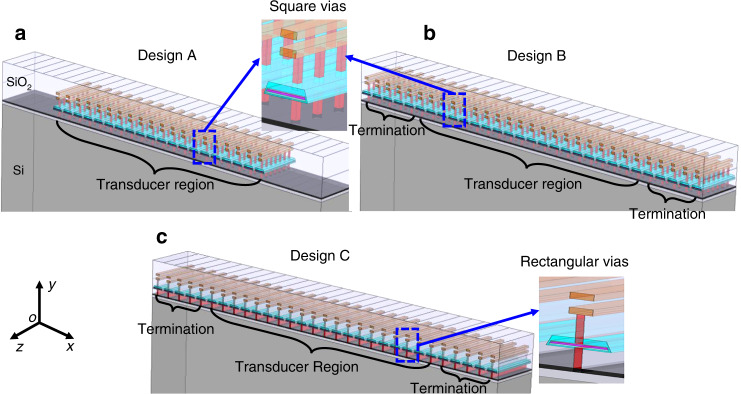
Schematics of resonator design. **a** with only main resonant cavity, **b** containing both main cavity and two terminations at the sides, and **c** with main cavity, terminations and traditional square vias replaced by rectangular vias

To optimize lateral confinement of the acoustic mode, termination of the acoustic waveguide in the plane of the wafer must be carefully considered. In Design A (Fig. 2a), the resonator is abruptly terminated at either end of the transducer region, leading to larger impedance mismatch, and subsequent scattering loss. In Design B (Fig. 2b), dummy FeCAP unit cells are added to either end of the transducers. These elements are spaced with the same period as the FeCAP transducer region. In this way, the boundaries of the transducers are extended towards the ends and the scattering losses inside the transducer region can be minimized.

The waveguided modes are optimized based on 2D analysis, assuming infinite uniform geometry in the transverse direction ($\overrightarrow {oz}$). In order to make the fabricated device match most closely with this approximation, one would maintain the structure in $\overrightarrow {oz}$ direction as continuous as possible ensuring high Q and minimization of spurious modes. However, as is traditionally found in IC technology, discrete-block vias are used to electrically route the FeCAPs to the first metal layer. Instead, in certain permutations of the FeCAP resonator, discrete (“square”) vias are replaced with continuous, rectangular, “wall-like” vias. to avoid scattering losses along $\overrightarrow {oz}$ direction, as shown in Fig. 2c.

**Fig. 3:**
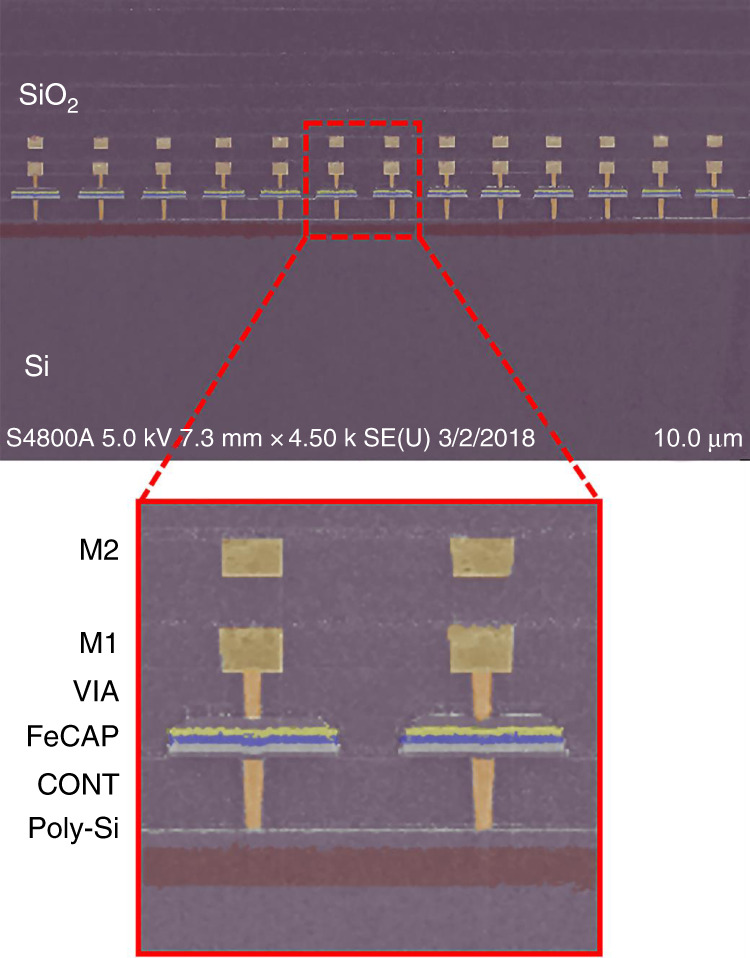
Cross sectional scanning electron micrograph of the CMOS-MEMS FeCAP-based resonator^30^

The corresponding scanning electron micrograph (SEM) of a side view schematic of the proposed resonator is plotted in Fig. 3, showing an array of trapezoidal FeCAPs each spanning 1.4 μm in length, connected to Cu strips by W vias^30^. The resonator consists of 20 transducers alternating with 2 μm periodicity to form the resonant cavity. The resonator has an overall footprint of 108 µm by 7 µm.

## Experimental results and discussion

**Fig. 4:**
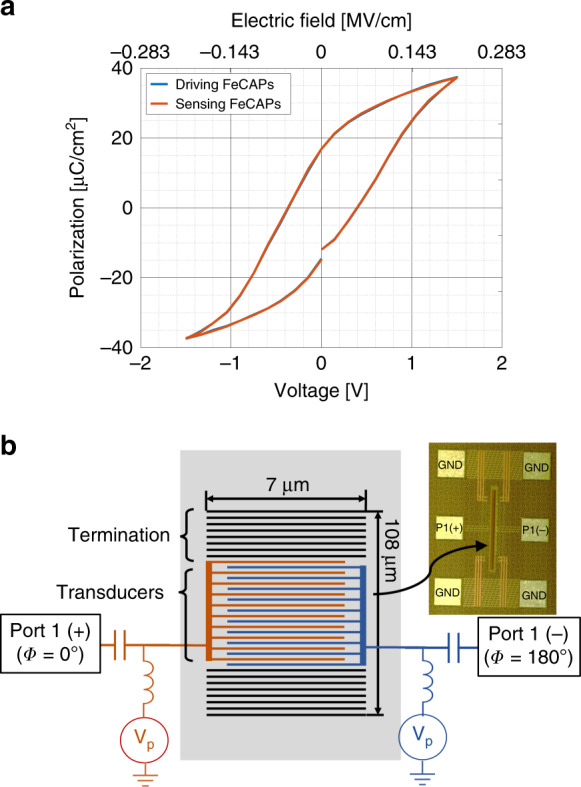
For efficient piezoelectric transduction, a polarization voltage V_P_ between −1.5 V and 1.5 V is applied across the device. ^30^. **a** Measured hysteresis of ferroelectric PZT FeCAP polarization. **b** Experimental setup to characterize the FeCAP resonator, including a schematic of the port connection to the transducers. Electrically isolated FeCAPs on either side of these transducers (black) are used to terminate the resonance cavity and provide in-plane elastic confinement. Inset shows an optical micrograph of the device

The ferroelectric properties of the FeCAP were first characterized to investigate PZT film behavior. Polarization-Electric field (P–E) measurement was carried out on a Radiant Technologies RT66C ferroelectric tester, shown in Fig. 4a. The operational voltages for the FeCAPs are limited between −1.5 V and 1.5 V by dielectric leakage. A coercive voltage of ±0.3 V is obtained with remnant polarization of 16.86 μC/cm^2^. The saturation polarization is 38 μC/cm^2^.

As previously noted, the entire device consists of 20 FeCAPs transducers. This gives a total FeCAP area of 196 µm^2^ on the transducers. The experimental setup is shown in Fig. 4b.

To enforce the excitation of the desired mode at the Brillouin zone edge ($k = \pi /a$), a differential signal was applied between alternating transducers. Differential 1-port RF measurement was performed in ambient pressure and temperature using a Keysight N5225A PNA. The poling voltage was provided by two Keithley 2400 source measurement units (SMUs), connected to ports 1(+) and 1(−) through bias tees. All transducers are biased at same poling voltage (V_p_). The RF power is −10 dBm which corresponds to a peak-to-peak voltage of 200 mV. The IF bandwidth (IFBW) is 500 Hz. The differential S-parameters can be obtained by the following equation

2 $$S_{dd11} = (S_{11} + S_{22} - S_{12} - S_{21})/2$$

**Fig. 5:**
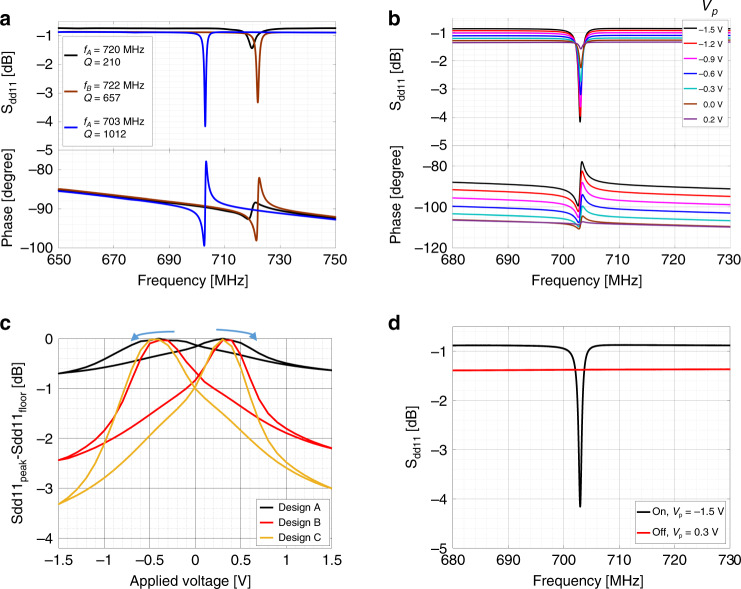
RF characterization of the FeCAP resonators. **a** Comparison of S_21_ for all the designs **a**–**c** **b** Measured frequency response of design C. **c** Due to the ferroelectric hysteresis, the S_21_ magnitude demonstrates ‘butterfly’-shaped variations with changing poling voltage. **d** At coercive voltages of ±0.3 V, the FeCAP transducer can be completely switched off

Figure 5 summarizes the measured RF response of the FeCAP resonators. We first consider resonator Design A, a waveguided resonance is observed at 720 MHz with Q of 210 (black line in Fig. 5a). For Design B, the same resonance mode is found at 722 MHz but Q increased to 657 (brown line in Fig. 5a). Then, due to mass-loading from the added W volume, the resonance frequency for Design C shifts down to 703 MHz and Q increases to 1012. With an *f*·Q product of 7.11 × 10^11^ (blue line in Fig. 5a), the performance is 1.6× higher than the most state-of-the-art PZT resonators. In addition, the resonator is monolithically integrable with CMOS platform^10^.

The dependence of *S*_*dd*11_ on poling voltage is shown in Fig. 5b varying *V*_*p*_ from −1.5 V to 0.2 V on both port 1 (+) and port 1 (−). The resulting ‘butterfly’-shaped *S*_*dd*11_ magnitude variation at resonance with poling voltage is shown in Fig. 5c. The magnitude of *S*_*dd*11_ reaches a minimum when all the transducers are biased at ±1.5 V. Additionally, piezoelectric transduction is suppressed when the device is biased at the FeCAP coercive voltage of ±0.3 V (Fig. 5d).

**Fig. 6:**
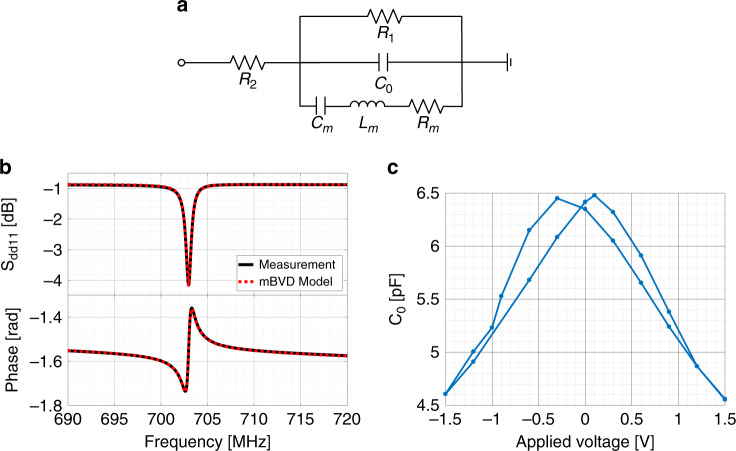
FeCAP resonator modeling. **a** Equivalent circuit of FeCAP resonator based on modified Butterworth-Van-Dyke model **b** S_dd11_ of mBVD model and the measurement for the resonator with extensions and rectangular vias. **c** Measured C_0_ variation with respect to the changing poling voltage

A small signal equivalent circuit of the FeCAP resonator based on a modified Butterworth-Van-Dyke (mBVD)^31^ is presented in Fig. 6a, consisting of a mechanical resonance branch which includes a motional resistance (*R*_*m*_), motional inductance (*L*_*m*_), and motional capacitance (*C*_*m*_), in parallel with *C*_0_. Here, *C*_0_ is defined as the geometric capacitance of the structure which is valid for a fixed poling voltage. In the meantime, *R*_1_ and *R*_2_ model any resistive losses from leakage in the FeCAP and routing, respectively. The equivalent circuit parameters fitted to the measured data for design C under a poling voltage of −1.5 V is shown in Fig. 6b. The corresponding circuit parameters is summarized in Table 1.

**Table 1:** Performance of resonator Design C

Parameters	Value	Unit
*R*_*m*_	134.5	Ω
*L*_*m*_	30.8	μH
*C*_*m*_	1.7	fF
*C*_0_	4.5	pF
*R*_1_	1959.8	Ω
*R*_2_	3.8	Ω
*Q*	1012	−
*K*^2^	0.047	−

The electromechanical coupling coefficient can be extracted as:^32^

3 $$k^2 = \frac{{\pi ^2}}{8}\frac{{C_m}}{{C_0}} = 0.047{\mathrm{\% }}$$

Under the full bias sweep, *C*_0_ varies between 4.6 pF and 6.5 pF as demonstrated in Fig. 6c.

Thermal stability of resonance frequency was characterized for the FeCAP resonators over a temperature range from 23 to 90 °C. The resonant frequency shift with respect to the temperature variation was summarized in ref. ^32^ and the Temperature Coefficient of Frequency was extracted as:^32^

4 $$TCF = \frac{1}{{f_0}}\frac{{\partial f}}{{\partial T}} = - 58.1 \pm 4.6\;\mathrm{ppm}/^\circ{\mathrm{C}}$$

This measured temperature sensitivity matches well to the predicted values based on the Temperature Coefficient of Young’s Modulus ($TCE = \Delta E/\Delta T$) of the constituent materials in the CMOS BEOL^32^.

## Conclusions

This work demonstrates a new class of ferroelectric-transduced RF MEMS resonators embedded seamlessly in CMOS, leveraging TI’s 130 nm FeRAM technology. The magnitude of the electromechanical response can be tuned by varying poling voltages on the transducers. Due to the hysteretic effect of the ferroelectric material, the magnitude of $S_{dd11}$ exhibit a hysteresis response with respect to changing poling voltages on the ports. The maximum *Q* of 1012 is obtained with optimization of vertical and lateral confinement of the acoustic mode in the CMOS stack. This corresponds to an *f*·*Q* of 7.11 × 10^11^.

**Fig. 7:**
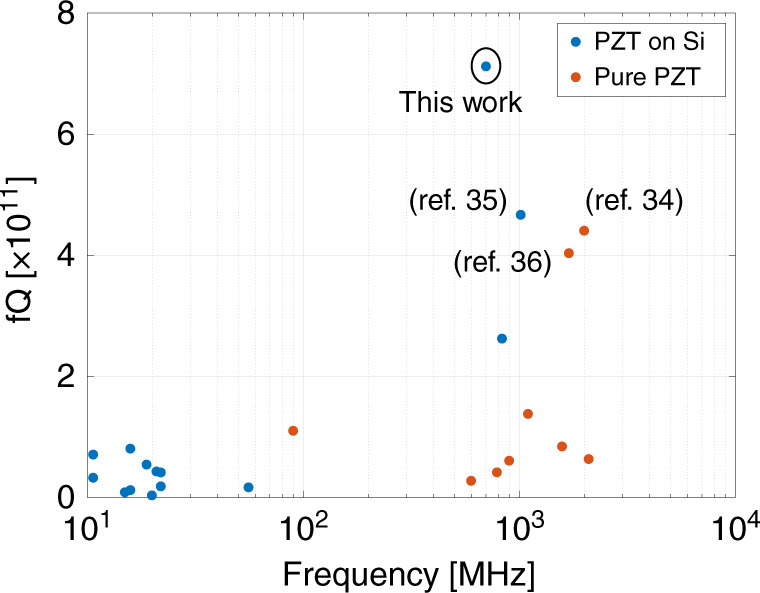
Comparison of *f*·Q with previous work

Figure 7 has summarized the *f·Q* for the previous work on both pure-PZT resonators (red dots), as well as the PZT-on-Si resonator (blue dots). This performance is 1.6× higher than state-of-the-art PZT resonators with the additional benefit of CMOS integration^33–36^.

The PZT material itself is typically acoustically lossy at radio frequencies. However, in this manuscript, the thickness of the PZT thin film is only about 70 nm while the entire capacitor is about 500 nm, and the mode extends as an evanescent tail beyond the FeCAP. From the mode shape diagram in Fig. 1c, the resonance volumetric ratio of PZT to non-PZT material (with 95% strain energy concentration) is about 1%. This will boost Q relative to pure-PZT devices due to the lower viscoelastic losses of surrounding materials. However, the higher Q also trades off performance with k^2^, which is reduced substantially (0.047%) relative to pure PZT resonators.

Suppression of electromechanical transduction is demonstrated when FeCAPs are biased at their coercive voltage of ±0.3 V. The extracted TCF is −58.1 ± 4.6 ppm/°C. Therefore, these devices provide a platform for applications including but not limited to RF components, timing, sensing, imaging, with CMOS integration.

## Supplementary information

Resonator Design Process

## References

[1] Chen CY, Li MH, Li CS, Li SS (2014). Design and characterization of mechanically coupled CMOS-MEMS filters for channel-select applications. Sens. Actuators A: Phys..

[2] Naify CJ, Rogers JS, Guild MD, Rohde CA, Orris GJ (2016). Evaluation of the resolution of a metamaterial acoustic leaky wave antenna. J. Acoust. Soc. Am..

[3] Sobreviela, G., Riverola, M., Torres, F., Uranga, A. & Barniol, N. in TRANSDUCERS 2017 − 19th International Conference on Solid-State Sensors, Actuators and Microsystems 1943–1946 (2017).

[4] Antonio, D., Zanette, D. H. & López, D. Frequency stabilization in nonlinear micromechanical oscillators. *Nat. Commun*. **3**, 806 (2012). 10.1038/ncomms1813 22549835

[5] Chen C, Zanette DH, Czaplewski DA, Shaw S, López D (2017). Direct observation of coherent energy transfer in nonlinear micromechanical oscillators. Nat. Commun..

[6] Huang YJ (2013). A CMOS cantilever-based label-free DNA SoC with improved sensitivity for hepatitis B virus detection. IEEE Trans. Biomed. Circuits Syst..

[7] Ahmed M (2017). Integrated CMOS-MEMS flow sensor with high sensitivity and large flow range. IEEE Sens. J..

[8] Li Q (2018). 0.04 degree-per-hour MEMS disk resonator gyroscope with high-quality factor (510 k) and long decaying time constant (74.9 s). Microsyst. Nanoeng..

[9] Kuo, J. C., Hoople, J. T., Abdelmejeed, M., Abdel-Moneum, M. & Lal, A. in *Proceedings of the IEEE International Conference on Micro Electro Mechanical Systems* (MEMS) 9–12 (2017).

[10] Bhugra, H. & Piazza, G. Piezoelectric MEMS Resonators. (2017).

[11] Weinstein D, Bhave SA (2010). The resonant body transistor. Nano Lett..

[12] Bahr, B. & Weinstein, D. in Solid-State Sensors, Actuators, and Microsystems Workshop 88–91 (2016).

[13] Bahr B, He Y, Krivokapic Z, Banna S, Weinstein D (2018). 32GHz resonant-fin transistors in 14 nm FinFET technology. Dig. Tech. Pap. - IEEE Int. Solid-State Circuits Conf..

[14] Bahr B, Marathe R, Weinstein D (2015). Theory and design of phononic crystals for unreleased CMOS-MEMS resonant body transistors. J. Microelectromechanical Syst..

[15] Kasap, S. & Capper, P. Springer Handbook of Electronic and Photonic Materials. Springer (2017).

[16] Gao, P. et al. Revealing the role of defects in ferroelectric switching with atomic resolution. *Nat. Commun*. **2**, 591 (2011). 10.1038/ncomms1600 22186887

[17] Khan AI (2015). Negative capacitance in a ferroelectric capacitor. Nat. Mater..

[18] Dubourdieu C (2013). Switching of ferroelectric polarization in epitaxial BaTiO3 films on silicon without a conducting bottom electrode. Nat. Nanotechnol..

[19] Valasek J (1921). Piezo-electric and allied phenomena in Rochelle salt. Phys. Rev..

[20] Hayashi T (2002). A novel stack capacitor cell for high density FeRAM compatible with CMOS logic. Dig. Int. Electron Devices Meet..

[21] Bertotti, G., Mayergoyz, I. D., Durin, G. & Zapperi, S. The Science of Hysteresis 2, (2006).

[22] Forsbergh PW (1949). Domain structures and phase transitions in barium titanate. Phys. Rev..

[23] Fu W (2011). Ferroelectric gated electrical transport in CdS nanotetrapods. Nano Lett..

[24] Gao J (2017). Enhancing dielectric permittivity for energy-storage devices through tricritical phenomenon. Sci. Rep..

[25] Jiang W, Cao W (2000). Nonlinear properties of lead zirconate-titanate piezoceramics. J. Appl. Phys..

[26] Ghosez P, Rabe KM (2000). Microscopic model of ferroelectricity in stress-free PbTiO3 ultrathin films. Appl. Phys. Lett..

[27] Müller, J. et al. Ferroelectricity in HfO2 enables nonvolatile data storage in 28 nm HKMG. Dig. Tech. Pap. - Symp. VLSI Technol. 25–26 (2012).

[28] Muller, J. et al. Ferroelectric hafnium oxide: A CMOS-compatible and highly scalable approach to future ferroelectric memories. Tech. Dig. - Int. Electron Devices Meet. IEDM 10.8.1-10.8.4 (2013).

[29] Udayakumar KR (2008). Manufacturable high-density 8 Mbit one transistor-one capacitor embedded ferroelectric random access memory. Jpn J. Appl. Phys..

[30] He, Y., Bahr B., Si, M., Ye, P. & Weinstein, D. Switchable Mechanical Resonance Induced by Hysteretic Piezoelectricity in Ferroelectric Capacitors. Transducer 2019 – The 20th International Conference on Solid-State Sensors, Actuators and Microsystems (2019).

[31] Larson, J. D., Bradley, P. D., Wartenberg, S. and Ruby, R. C., Modified Butterworth-Van Dyke circuit for FBAR resonators and automated measurement system. 2000 IEEE Ultrasonics Symposium. Proceedings. An International Symposium (Cat. No. 00CH37121) (Vol. 1, pp. 863-868).October, 2000.

[32] He, Y., Bahr B., & Weinstein, D. A Ferroelectric Capacitor (FeCAP) Based Unreleased Resonator. Hilton Head 2018 – A Solid-State Sensors, Actuators and Microsystems Workshop, 71–74, 2018.

[33] Piazza G, Stephanou PJ, Pisano AP (2006). Piezoelectric aluminum nitride vibrating contour-mode MEMS resonators. J. Microelectromechanical Syst..

[34] Schreiter M, Gabl R, Pitzer D, Primig R, Wersing W (2004). Electro-acoustic hysteresis behaviour of PZT thin film bulk acoustic resonators. J. Eur. Ceram. Soc..

[35] Chandrahalim, H., Bhave, S. A., Polcawich, R. G., Pulskamp, J. S. and Kaul, R., PZT transduced high-overtone width-extensional resonators above 1 GHz. 2009 IEEE International Ultrasonics Symposium, pp. 2145–2148, 2009.

[36] Hanajima N (1997). Ultrasonic properties of lead zirconate titanate thin films in UHF-SHF range. Jpn J. Appl. Phys., Part 1: Regul. Pap. Short. Notes Rev. Pap..

