# Supplementary material for: A tunable ferroelectric based unreleased RF resonator
Source: Microsyst Nanoeng. 2020 Jan 13;6:8. doi: 10.1038/s41378-019-0110-1 (PMC8433143; doi:10.1038/s41378-019-0110-1)
Supplement: Supplementary file 1 — Resonator Design Process [file 41378_2019_110_MOESM1_ESM.pdf]

Supplementary Information

Resonator Design Process:

Two-dimensional finite element analysis is performed by COMSOL Multiphysics® to investigate and optimize resonance modes in the FeRAM CMOS stack. As noted in the article, Floquet periodic boundary conditions are applied at the left and right boundaries, while perfectly matched layers (PMLs) are applied at the top and the bottom of the device so as to elliminate the elastic wave reflections from there. By searching the eigenmodes along the first Brillouin zone, we can obtain the dispersion relations of acoustic waves in the CMOS stack.

Limited by the technology, a maximum of 5 metal layers are used in the process. While lithographic dimensions can be changed within the constraints of the design rules, materials and thicknesses of the layers are predetermined by the technology. Practically speaking, the first metal layer must be reserved for electrical routing to the transducers, and must therefore be omitted from the BEOL reflector design. Based on these design constraints, we optimize the following parameters to maximize energy confinement in the resonance cavity:

- Number of metal layers used in the BEOL reflector
- Width of each metal layer in the *x*-direction
- Lattice constant *a* in the *x*-direction

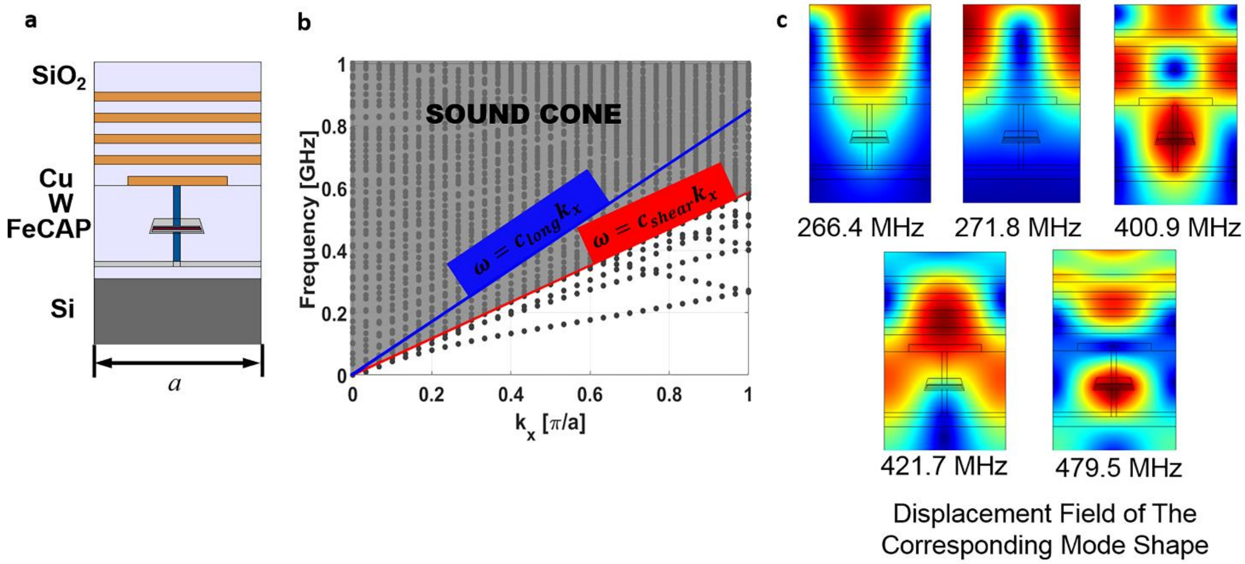

Fig. S1 a Schematic of FeCAP acoustic waveguide unit cell with 5 metal layers. **b** Dispersion relation for the device. **c** The mode shapes of localized modes found along $k_x = \pi/a$.

Mode optimization begins with a range of devices using 5 metal layers at the BEOL. Apart from the first metal layer, all the other layers are continuous across the FeCAP area (Figure S1(a)). It is assumed that the

length of the trapizoidal FeCAP is 1.4 μm and the length of the lattice constant a is 5 μm. The dispersion relation, obtained through eigenmode analysis in COMSOL, is shown in Fig. S1(b). The blue and red lines present the longitudinal and shear sound lines, respectively. Several discrete modes exist below the shear sound line, indicating modes which are slow enough to be guided laterally in the CMOS stack. However, according to the stress distribution of these guided modes along $k_x = \pi/a$, only the modes at 400.9 MHz and 479.5MHz are coupled efficiently to the FeCAP transducer. These mode shapes also indicate the energy confined in the FeCAP is coupled with the metal layers above it, corresponding to acoustic loss. Additionally, the localized modes are very close to the shear sound line, indicating an opportunity for scattering into the bulk Si corresponding to lower quality factor.

To push the strain energy downwards closer to the FeCAP, the number of metal layers is reduced to two. The schematic of the device is shown in Fig. S2(a), with corresponding dispersion relation in Fig. S2(b). Five localized modes are visible along $k_x = \pi/a$ below shear sound line. Their displacement field is plotted in Fig. S2(c). In this case, only the mode at 433.4 MHz results in a large overlap between the strain energy and the transducer area. This is a necessary characteristic for high efficiency electromechanical coupling for drive and sense. However, the energy is still coupled with part of the SiO_2 layers and it is not fully confined within the FeCAP area. To simplify the optimization process taking into account technology limits, the length of the FeCAP is fixed at 1.4 μm. Sweeping the geometry of the metal layers, it is found that a metal length of 600 nm provides the best confinement.

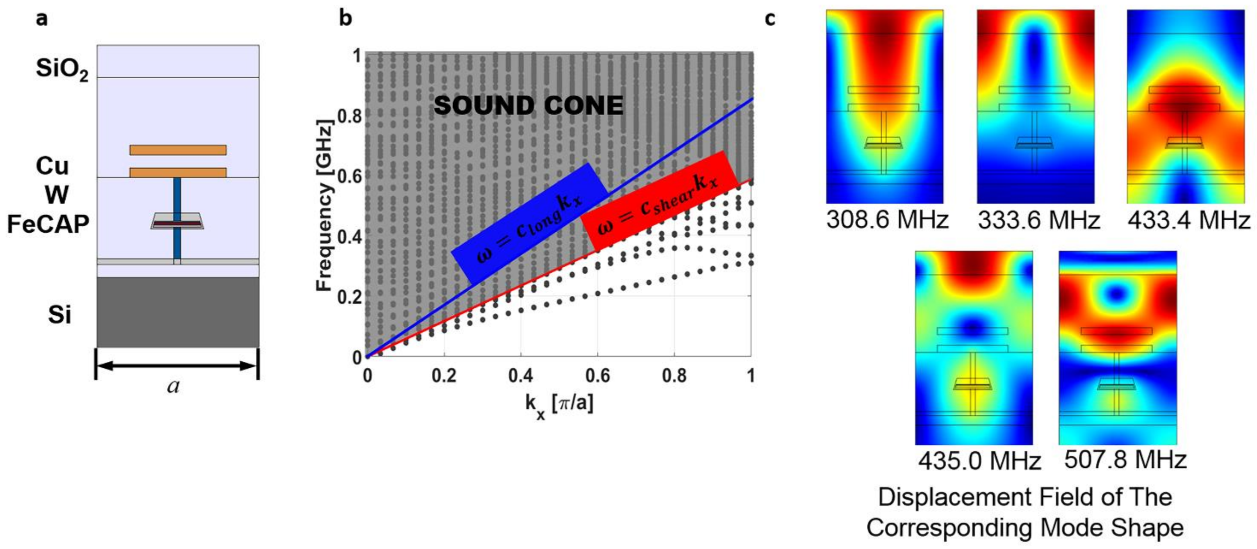

Fig. S2 a Schematic of FeCAP unit cell with two metal layers. **b** Dispersion relation for the device with two metal layers. **c** The mode shape of localized modes found along $k_x = \pi/a$.

To further enhance the energy confinement, the authors investigated the effect of decreasing the unit cell length a. This is because, the sound line is expressed as $\omega = c \cdot k$, where c is the acoustic wave velocity of a given material and k is the wavenumber. Normalizing k with $\frac{\pi}{a}$ and we obtain

$$f = \frac{\omega}{2\pi} = \frac{c}{2a}k\,.$$

Thus, the slope of the sound line is inversely proportional with a. By decreasing a, the resonance mode frequency is increased and when a=2 um (lithographic limit of the techonology), and the distance between the confined mode and the sound line reaches maximum. This provides lower probability of mode scattering into the bulk Si and indicates highest energy confinement.

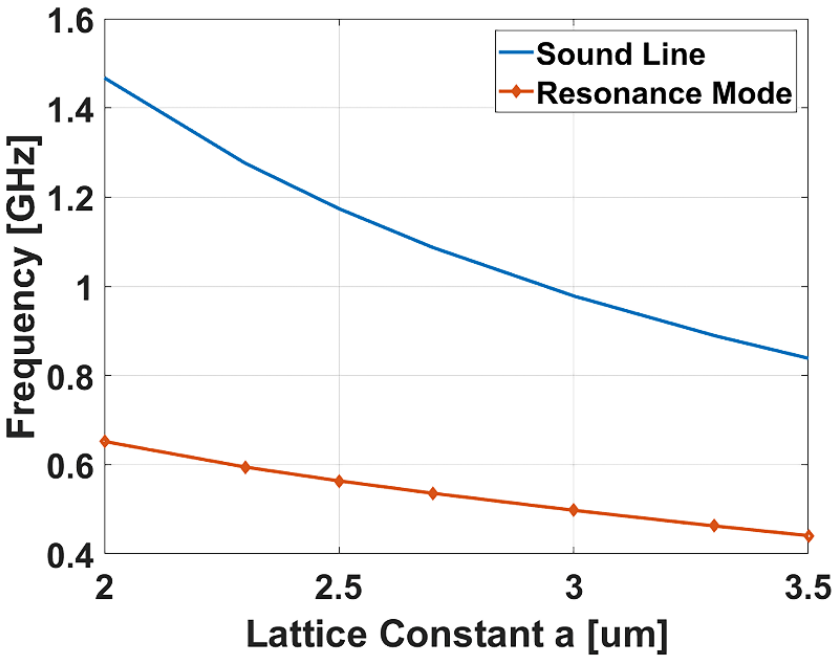

Fig. S3 Dependence of the acoustic mode frequency on the changing lattice constant a. As lattice constant reduces, the distance between the localized mode and the sound line increases.

To conclude, the metal width of 600 nm for metals 1 and 2, with lattice cosntant a = 2 um provides a combination of large acoustic confinment with maximum overlap of elastic energy in the FeCAP transducer, within the lithography constraints of the TI E035 technology. The corresponding dispersion relation is shown in Fig. 2 of the manuscript.
